# The different associations between platelet distribution width and hypertension subtypes in males and females

**DOI:** 10.1042/BSR20201747

**Published:** 2020-11-19

**Authors:** Tingwei Li, Jiahui Jin, Zhaowei Meng, Wenjuan Zhang, Yongle Li, Xuefang Yu, Xin Du, Ming Liu, Qing Zhang, Ying Gao, Kun Song, Xing Wang, Yaguang Fan, Yan Wang

**Affiliations:** 1 Department of Nuclear Medicine, Tianjin Medical University General Hospital, Tianjin, PR China; 2 Tianjin University of Traditional Chinese Medicine, Jian Kang Chan Ye Yuan, Jinghai District, Tianjin, PR China; 3 Department of Cardiology, Tianjin Medical University General Hospital, Tianjin, PR China; 4 Department of Endocrinology and Metabolism, Tianjin Medical University General Hospital, Tianjin, PR China; 5 Department of Health Management, Tianjin Medical University General Hospital, Tianjin, PR China; 6 Tianjin Key Laboratory of Lung Cancer Metastasis and Tumor Microenvironment, Tianjin Lung Cancer Institute, Tianjin Medical University General Hospital, Tianjin, PR China

**Keywords:** age, hypertension subtype, hypertension, platelet distribution width, platelet, sex

## Abstract

The prevalence of hypertension has increased rapidly in recent years. Currently, increasing attention has been paid to the relationship between hypertension and platelet abnormalities. As a simple and available platelet parameter, platelet distribution width (PDW) can reflect platelet abnormalities and further reflect the risk of thrombotic diseases. However, the views on PDW and hypertension are controversial at present studies. Hence, we aimed to find the associations between PDW and hypertension subtypes in the present study. A total of 73,469 participants (44,665 males and 28,804 females) were enrolled. We found that PDW was a risk factor for isolated systolic hypertension (ISH), and the risk of ISH increased with PDW quartiles among women. In men, high PDW might be a risk factor for isolated diastolic hypertension and systolic–diastolic hypertension.

## Introduction

Hypertension is threatening human health worldwide and poses great challenges to public health [1]. In the past few decades, the prevalence of hypertension has dramatically increased in China, causing severe hypertension complications, such as stroke and heart disease, which are leading factors of death and disability-adjusted life-years [2]. Although we have made some progress in the management of hypertension, the shortcomings in terms of the awareness, treatment, and control of hypertension are worrying [3]. More importantly, long-term exposure to high blood pressure results in vascular extracellular remodeling, which is closely associated with damage to the vasculature, myocardium, and kidneys [4]. Different types of hypertension possess special clinical manifestations and characteristics. Ahmed et al. [5] mentioned that increased arterial stiffness and decreased arterial compliance contributed to isolated systolic hypertension (ISH), and individuals with high systolic blood pressure are more prone to coronary heart disease, renal failure, and stroke, especially among people over 65 years of age. Kim et al. [6] mentioned that ISH was closely associated with the risk of stroke and coronary heart disease. Arima et al. [7] noted that all hypertensive subtypes were observed to have various cardiovascular diseases, such as coronary heart disease, ischemic stroke, and hemorrhagic stroke. Li et al. [8] concluded that isolated diastolic hypertension (IDH) and systolic–diastolic hypertension (SDH) were significantly associated with an increased risk of cardiovascular diseases. Nielsen et al. [9] posited that systolic blood pressure and diastolic blood pressure were not as effective in predicting the risk of future hypertension complications and that the former would be a better predictor. Lip et al. [10] suggested that ISH and SDH presented no significant differences in the prothrombotic state, endothelial dysfunction, or risk of stroke and heart attack.

Platelets are blood cells without nuclei derived from bone marrow-located megakaryocytes and play a critical role in the process of blood coagulation [11]. Platelet distribution width (PDW) is a common platelet parameter that has been used to assess the volume heterogeneity of platelets. High PDW indicates that the volume of platelets is nonuniform [12]. More importantly, PDW is a marker of platelet activation and presents a positive correlation with platelet activation, which contributes to thrombotic disease [13]. Increased platelet activity is considered a significant change associated with hypertension and is a potential cause of hypertension complications [14].

In the current literature, many studies have focused on the relationship between mean platelet volume (MPV) and hypertension [15–17]. Studies concerning PDW and blood pressure and even hypertension subtypes are quite rare and controversial. A study from Gregory et al. [10] concluded that ISH was closely related to prothrombotic and endothelial dysfunction, and a similar phenomenon can also be seen in SDH. Research data from Abudesimu et al. [18] showed that the difference in PDW among patients with various types of hypertension is not significant; however, ISH, compared with normal blood pressure, was a risk factor for abnormal PDW. Research by Yang et al. [19] mentioned that PDW was negatively associated with systolic blood pressure (SBP) and that there was no association between PDW and diastolic blood pressure (DBP). Thus, based on blood pressure level, we sorted hypertension into three subtypes, ISH, IDH and SDH, by sex. We aimed to investigate the relationship, if any, between hypertension subtypes and PDW by sex.

## Methods

### Participants and agreement

A total of 73,469 participants (44,665 males and 28,804 females) were enrolled who underwent a physical examination at the Tianjin Medical University General Hospital between 2007 and 2015. Exclusion criteria were as follows: a history of hematological, liver, kidney or oncological diseases, smoked, consumed alcohol and used medicine that might affect blood pressure and blood parameters. All participants were informed and written consent was obtained. The institutional review board and ethics committee of Tianjin Medical University General Hospital approved the present study, and a detailed protocol was reported in our previous publications [20–22].

### Data collection

The investigation of personal details was accomplished via a questionnaire. Routine physical measurements of height, weight, body mass index (BMI), and abdominal circumference were performed. Three blood pressure measurements were performed using a standard mercury sphygmomanometer by a trained nurse in a quiet space after participants rested for at least 15 min, then, the average was taken. Blood samples were obtained after participants had fasted for at least 8 h. The samples used for routine blood tests were collected in vacuum blood collection tubes with ethylenediaminetetraacetic acid and analyzed using a hemocytometer analyzer (Sysmex Corporation, Kobe, Japan). Other samples used for blood lipid and renal function tests were collected in vacuum blood collection tubes with inert separating glue and analyzed by an autoanalyzer (Hitachi Model 7600 analyzer, Hitachi, Tokyo, Japan).

### Laboratory reference ranges and definitions

The laboratory reference ranges were as follows: creatinine (Cr) 44–115 μmol/l, uric acid (UA) 62–133 μmol/l, total cholesterol (TC) 3.59–5.17 mmol/l, triglycerides (TGs) 0.57–1.71 mmol/l, high-density lipoprotein (HDL) 0.8–2.2 mmol/l, low-density lipoprotein (LDL) 1.33–3.36 mmol/l, glucose (GLU) 3.6–5.8 mmol/l, and PDW 9.0–17.0 fl.

The hypertension subtypes were defined according to SBP and DBP levels as follows: ISH, SBP ≥ 140 mmHg and DBP < 90 mmHg; IDH, SBP < 140 mmHg and DBP ≥ 90 mmHg; SDH, SBP ≥ 140 mmHg and DBP ≥ 90 mmHg.

### Statistical analysis

The tool used for the analysis was SPSS (SPSS version 24.0, Chicago, IL). All data were separated into sex and hypertension subtype groups. Continuous variables are reported as the mean ± standard deviation. A comparison of continuous variables between different groups was accomplished by independent sample *t*-tests. The chi-square test was applied to compare prevalence differences between the sexes. The correlation of hypertension subtypes with different parameters was analyzed by logistic regression. The PDW was divided equally into four parts. As covariates were different, three models were created. The odds ratios (ORs) were calculated by binary logistic regression for hypertension subtypes with 95% confidence intervals. The difference was significant when *P* was <0.05.

## Results

**Figure 1:**
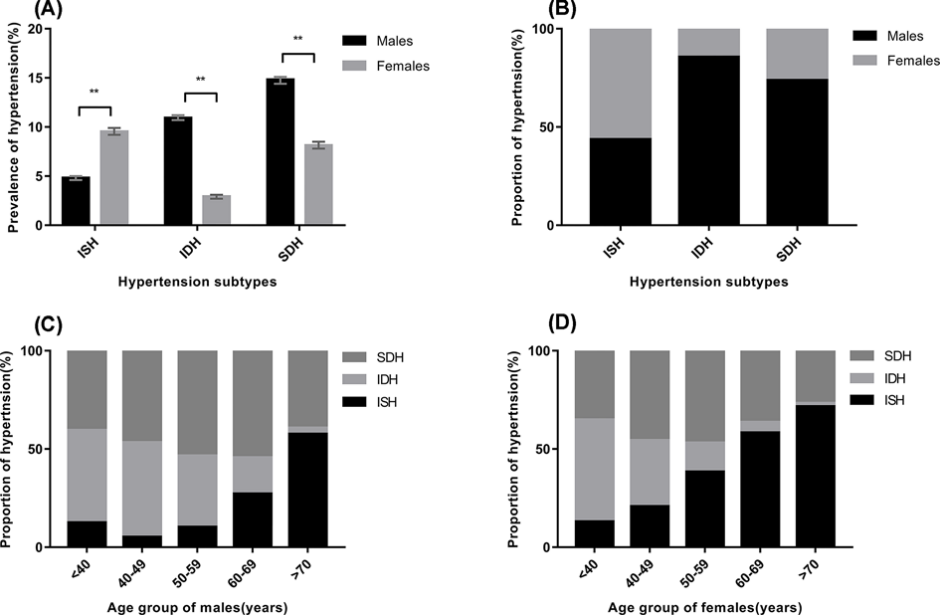
The prevalence and proportion of different hypertension subtypes (**A**) The prevalence, with 95% confidence interval, of different hypertension subtypes by sex (analyzed by chi-square test between males and females, **P<0.01). (**B**) The proportion of different hypertension subtypes by sex. (**C** and** D**) The proportion of different hypertension subtypes in different age groups.

The prevalence of ISH in females was higher than that in males. However, IDH and SDH might occur more often in males (Figure 1A). Women accounted for more than half of patients with ISH; in contrast, among patients with IDH and SDH, the proportion of men far exceeded the proportion of women (Figure 1B). Among females with hypertension, the proportion of ISH gradually increased with age, and it exceeded half of the population over 60 years. Moreover, the proportion of individuals with IDH decreased with age. Except for in the group older than 70 years old, individuals with IDH and SDH accounted for most of the male hypertensive population (Figure 1C,D).

**Figure 2:**
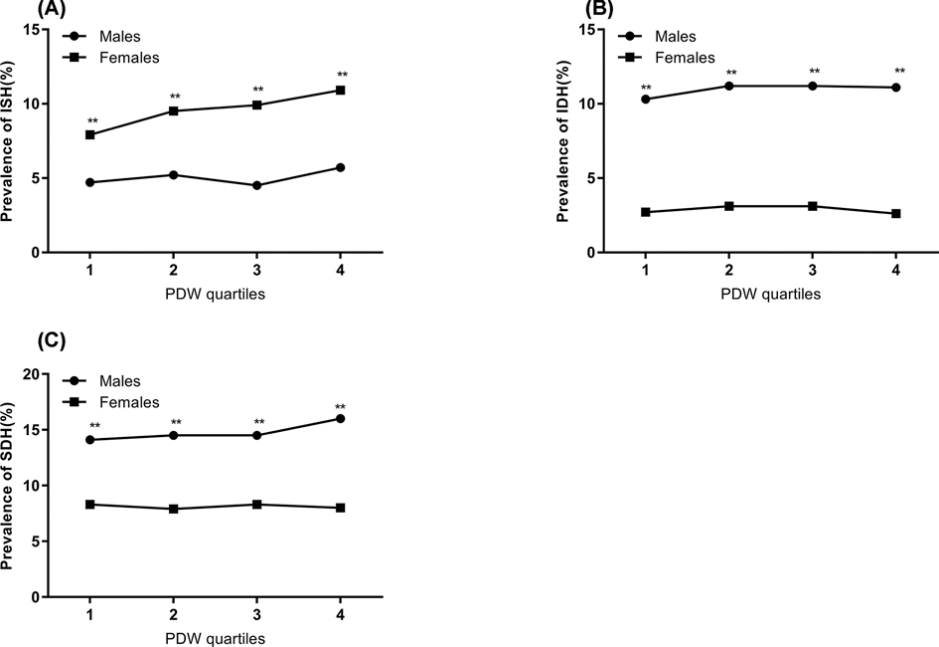
The prevalence of different hypertension subtypes by PDW quartile (**A**) The prevalence of isolated systolic hypertension (ISH) by platelet distribution width (PDW) quartile. (**B**) The prevalence of isolated diastolic hypertension (IDH) by PDW quartile. (**C**) The prevalence of systolic diastolic hypertension (SDH) by PDW quartile. Analyzed by chi-square test between males and females in each quartile, **P<0.01.

Compared with males, the prevalence of ISH was significantly higher among females. The prevalence of ISH tended to increase as the PDW quartile increased in females (*P*<0.01), but the differences between PDW quartiles were insignificant among males. Regarding the IDH subtype, the lines indicated that significant differences existed between males and females. Males had a higher prevalence than females, but the prevalence trend did not reach statistical significance in either sex. Regarding the SDH subtype, the condition was similar to the IDH subtype, but the trend of hypertension in males presented as a zigzag pattern (*P*<0.01; Figure 2).

Compared with females, males were younger. BMI coupled with waist circumference, which estimated the extent of obesity, revealed that males were more obese than females. In males, SBP and DBP were higher than in females. All hematologic indices except LDL showed significant differences between sexes. In addition to TC, HDL and PDW, other hematologic indices were also higher in males (Table 1).

**Table 1:** Characteristics by sex

Parameters	Males	Females	*T* value
Case number	44665	28804	
Age (years)	46.51±12.21	47.64±13.08	-11.841**
BMI (kg/m^2^)	25.88±3.21	23.96±3.47	76.882**
Waist circumference (cm)	89.48±8.85	78.89±9.61	153.01**
SBP (mmHg)	125.45±15.91	121.41±18.16	31.765**
DBP (mmHg)	81.06±11.06	74.81±10.25	76.934**
Cr (μmol/l)	79.35±11.13	59.83±9.46	245.877**
UA (μmol/l)	363.57±74.32	264.98±59.44	189.431**
TC (mmol/l)	5.09±0.91	5.19±0.98	-14.046**
TGs (mmol/l)	1.74±1.11	1.26±0.79	64.865**
HDL (mmol/l)	1.28±0.31	1.56±0.36	-112.647**
LDL (mmol/l)	3.07±0.80	3.07±0.86	-0.3
PDW (fl)	12.33±1.83	12.39±1.82	-4.692**
GLU (mmol/l)	5.26±0.89	5.05±0.73	34.119**

Abbreviations: body mass index (BMI), systolic blood pressure (SBP), diastolic blood pressure (DBP), creatinine (Cr), uric acid (UA), total cholesterol (TC), triglycerides (TGs), high-density lipoprotein (HDL), low-density lipoprotein (LDL), platelet distribution width (PDW), glucose (GLU). Analyzed by independent sample's t test.

** *P*<0.01.

Participants with hypertension subtypes were older than normotensive participants in both males and females. BMI and waist circumference revealed that elevated blood pressure and obesity were likely to exist simultaneously in both sexes. In males, the Cr between different hypertension subtypes and the normal blood pressure subgroup were similar. Different from HDL, the UA, TGs and PDW values of the IDH and SDH subgroups were higher than those of the normal blood pressure subgroups, and the differences between the ISH and normal subgroups were not significant. The values of TC, LDL and GLU were lower in the normal subgroup than in the three hypertension subgroups in the analysis of males. All hematologic parameters except Cr, HDL, and PDW showed higher levels in the three hypertension subgroups than in the normal blood pressure subgroup in the analysis of females. Compared with the normal blood pressure subgroup, the values of HDL in all hypertension subgroups were lower. The Cr of ISH and SDH groups as well as the PDW of the ISH group were higher than those in the normal subgroups (Table 2).

**Table 2:** Characteristics by different hypertension subtype

Sex	Parameters	Normal	ISH	IDH	SDH
Male	Age (years)	44.22±11.63	60.30±14.55**	47.40±9.03^†^	52.22±11.17^‡^
	BMI (kg/m^2^)	25.37±3.09	26.43±3.23**	26.85±2.98^†^	27.40±3.25^‡^
	Waist circumference (cm)	87.96±8.59	92.34±8.74**	92.04±8.05^†^	93.87±8.57^‡^
	SBP (mmHg)	117.83±10.09	146.67±8.59**	130.57±4.69^†^	150.70±10.95^‡^
	DBP (mmHg)	75.71±7.23	81.39±4.75**	92.16±3.37^†^	97.97±7.37^‡^
	Cr (μmol/l)	79.34±10.85	79.76±12.64	97.28±10.88	79.30±12.03
	UA (μmol/l)	360.04±72.48	357.07±77.59	375.50±76.42^†^	373.44±78.26^‡^
	TC (mmol/l)	5.01±0.90	5.21±0.90**	5.21±0.89^†^	5.34±0.93^‡^
	TGs (mmol/l)	1.65±1.04	1.67±0.97	2.01±1.21^†^	2.02±1.29^‡^
	HDL (mmol/l)	1.29±0.31	1.29±0.32	1.25±0.30^†^	1.27±0.31^‡^
	LDL (mmol/l)	3.02±0.79	3.19±0.80**	3.12±0.80^†^	3.23±0.83^‡^
	PDW (fl)	12.30±1.83	12.33±1.82	12.38±1.83^†^	12.43±1.85^‡^
	GLU (mmol/l)	5.14±0.97	5.65±1.12**	5.37±0.88^†^	5.61±1.08^‡^
Female	Age (years)	44.72±11.97	63.15±9.78**	49.33±9.59^†^	57.41±9.90^‡^
	BMI (kg/m^2^)	23.36±3.18	26.21±3.57**	25.30±3.35^†^	26.63±3.66^‡^
	Waist circumference (cm)	77.10±8.85	86.72±9.27**	81.60±8.43^†^	86.33±9.13^‡^
	SBP (mmHg)	114.39±11.53	149.34±10.17**	130.95±4.58^†^	153.93±12.05^‡^
	DBP (mmHg)	71.64±7.80	79.22±6.14**	91.48±2.72^†^	94.80±5.67^‡^
	Cr (μmol/l)	59.45±9.10	62.24±11.12**	59.54±9.22	60.81±10.41^‡^
	UA (μmol/l)	259.06±56.54	291.78±64.21**	271.58±60.14^†^	289.27±65.75^‡^
	TC (mmol/l)	5.08±0.95	5.71±0.95**	5.30±0.96^†^	5.66±0.95^‡^
	TGs (mmol/l)	1.16±0.72	1.64±0.85**	1.51±0.96^†^	1.69±0.97^‡^
	HDL (mmol/l)	1.58±0.36	1.49±0.35**	1.49±0.35^†^	1.48±0.34^‡^
	LDL (mmol/l)	2.98±0.83	3.50±0.86**	3.15±0.84^†^	3.44±0.85^‡^
	PDW (fl)	12.37±1.82	12.61±1.79**	12.33±1.76	12.40±1.87
	GLU (mmol/l)	4.95±0.62	5.51±0.99**	5.15±0.75^†^	5.43±0.93^‡^

Abbreviations: isolated systolic hypertension (ISH), isolated diastolic hypertension (IDH), systolic diastolic hypertension (SDH), body mass index (BMI), systolic blood pressure (SBP), diastolic blood pressure (DBP), creatinine (Cr), uric acid(UA), total cholesterol (TC), triglycerides (TG), high-density lipoprotein (HDL), low-density lipoprotein (LDL), platelet distribution width (PDW), glucose (GLU). Analyzed by independent sample’s *t* test.

** *P*<0.01 normal vs ISH groups;

† *P*<0.01 normal vs IDH groups;

‡ *P*<0.01 normal vs SDH groups

Except for UA, most of the parameters were associated with the development of some or all hypertension subtypes in males. In contrast, HDL might have been a protective factor. Similar results emerged among females. Most parameters, including age, BMI, waist circumference, Cr, UA, TC, TGs, LDL, PDW and GLU, had a negative effect on the development of different subtypes of hypertension. HDL also appeared to be a protective factor among females (Table 3).

**Table 3:** The likelihood of having different subtypes of hypertension according to different parameters

Sex	Parameters	ISH	IDH	SDH
		OR(CI)	OR(CI)	OR(CI)
Male	Age (years)	1.108(1.104-1.113) **	1.025(1.022-1.027) **	1.059(1.056-1.061) **
	BMI (kg/m2)	1.110(1.096-1.126) **	1.161(1.15-1.173) **	1.219(1.208-1.23) **
	Waist circumference (cm)	1.059(1.054-1.064) **	1.057(1.053-1.061) **	1.082(1.078-1.085) **
	Cr (μmol/l)	1.003(0.999-1.007)	1.000(0.997-1.002)	1.000(0.997-1.002)
	UA (μmol/l)	0.999(0.999-1.000)	1.003(1.002-1.003) **	1.002(1.002-1.003) **
	TC (mmol/l)	1.263(1.204-1.325) **	1.269(1.228-1.312) **	1.474(1.432-1.517) **
	TGs (mmol/l)	1.022(0.981-1.065)	1.292(1.262-1.323) **	1.297(1.27-1.325) **
	HDL (mmol/l)	0.990(0.859-1.140)	0.686(0.62-0.76) **	0.864(0.793-0.943) **
	LDL (mmol/l)	1.309(1.241-1.382) **	1.173(1.130-1.217) **	1.385(1.340-1.431) **
	PDW (fl)	1.010(0.986-1.034)	1.024(1.008-1.041) **	1.040(1.025-1.055) **
	GLU (mmol/l)	1.672(1.608-1.739) **	1.344(1.301-1.388) **	1.676(1.631-1.722) **
Female	Age (years)	1.142(1.137-1.147) **	1.032(1.026-1.038) **	1.092(1.088-1.096) **
	BMI (kg/m^2^)	1.264(1.249-1.278) **	1.177(1.155-1.200) **	1.297(1.281-1.314) **
	Waist circumference (cm)	1.113(1.108-1.118) **	1.054(1.046-1.062) **	1.109(1.104-1.114) **
	Cr (μmol/l)	1.030(1.026-1.034) **	1.001(0.994-1.009)	1.015(1.011-1.020) **
	UA (μmol/l)	1.009(1.008-1.009) **	1.004(1.002-1.005) **	1.008(1.007-1.009) **
	TC (mmol/l)	1.927(1.849-2.008) **	1.275(1.188-1.369) **	1.839(1.760-1.921) **
	TGs (mmol/l)	1.841(1.762-1.923) **	1.494(1.406-1.587) **	1.869(1.788-1.954) **
	HDL (mmol/l)	0.459(0.408-0.515) **	0.482(0.393-0.590) **	0.444(0.391-0.503) **
	LDL (mmol/l)	1.999(1.909-2.093) **	1.265(1.167-1.371) **	1.852(1.764-1.945) **
	PDW (fl)	1.073(1.051-1.096) **	0.988(0.951-1.027)	1.010(0.987-1.034)
	GLU (mmol/l)	2.314(2.206-2.428) **	1.482(1.363-1.611) **	2.217(2.023-2.236) **

Abbreviations: isolated systolic hypertension (ISH), isolated diastolic hypertension (IDH), systolic diastolic hypertension (SDH), body mass index (BMI), creatinine (Cr), uric acid (UA), total cholesterol (TC), triglycerides (TGs), high-density lipoprotein (HDL), low-density lipoprotein (LDL), platelet distribution width (PDW), glucose (GLU). Analyzed by binary logistic regression.

** *P*<0.01.

PDW quartile was designated as a categorical variable, with the lowest quartile used as the reference. As covariates were different, three models were created. Regarding ISH, the results varied by sex. In males, except for the results of quartile 2 in model 1 and model 2, we did not find significant outcomes by quartile. Among females, high PDW had a detrimental effect on ISH. OR values increased as PDW increased in the same models. Regarding IDH, the results in men showed that high PDW might indicate a higher possibility of developing IDH; however, the possibility did not increase with PDW quartiles continually and even disappeared in quartile 3 when covariates were added to the model. Among women, there were no significant OR values. Regarding SDH, in all models, quartile 4 of PDW had a disadvantageous impact on men, but the OR value decreased as the covariates added to the model increased. Similar impacts were observed among women in quartile 3 in model 2 and model 3 when we added age, BMI, waist circumference, Cr, UA, TC, TGs, LDL, HDL and GLU as covariates (Table 4).

**Table 4:** The OR(CI) of different subtypes of hypertension by PDW quartile

Sex	PDW quartile	PDW values	Model 1	Model 2	Model3
Male	ISH				
	quartile 1	PDW≤11.1(reference)			
	quartile 2	11.1<PDW≤12.1	1.130(1.001-1.275) *	1.154(1.011-1.317) *	1.128(0.998-1.289)
	quartile 3	12.1<PDW≤13.4	0.976(0.861-1.105)	0.963(0.841-1.102)	0.939(0.820-1.076)
	quartile 4	PDW>13.4	1.046(0.923-1.185)	1.131(0.988-1.296)	1.112(0.969-1.275)
	IDH				
	quartile 1	PDW≤11.1(reference)			
	quartile 2	11.1<PDW≤12.1	1.113(1.022-1.212) *	1.121(1.028-1.222) *	1.117(1.023-1.219) *
	quartile 3	12.1<PDW≤13.4	1.106(1.016-1.204) *	1.090(0.999-1.188)	1.071(0.981-1.168)
	quartile 4	PDW>13.4	1.120(1.027-1.220) *	1.119(1.025-1.221) *	1.095(1.002-1.197) *
	SDH				
	quartile 1	PDW≤11.1(reference)			
	quartile 2	11.1<PDW≤12.1	1.047(0.971-1.129)	1.044(0.963-1.132)	1.041(0.959-1.130)
	quartile 3	12.1<PDW≤13.4	1.041(0.965-1.122)	1.004(0.927-1.088)	0.985(0.908-1.069)
	quartile 4	PDW>13.4	1.179(1.094-1.270) **	1.166(1.077-1.263) **	1.147(1.058-1.245) **
Female	ISH				
	quartile 1	PDW≤11.2(reference)			
	quartile 2	11.2<PDW≤12.2	1.232(1.099-1.382) **	1.356(1.185-1.552) **	1.304(1.138-1.494) **
	quartile 3	12.2<PDW≤13.4	1.293(1.150-1.452) **	1.357(1.197-1.580) **	1.349(1.174-1.551) **
	quartile 4	PDW>13.4	1.424(1.272-1.593) **	1.409(1.232-1.611) **	1.370(1.197-1.569) **
	IDH				
	quartile 1	PDW≤11.2(reference)			
	quartile 2	11.2<PDW≤12.1	1.135(0.933-1.381)	1.186(0.973-1.446)	1.189(0.975-1.451)
	quartile 3	12.1<PDW≤13.4	1.160(0.957-1.405)	1.213(1.000-1.472)	1.214(0.999-1.474)
	quartile 4	PDW>13.4	0.987(0.807-1.207)	1.016(0.830-1.244)	1.010(0.824-1.238)
	SDH				
	quartile 1	PDW≤11.2(reference)			
	quartile 2	11.2<PDW≤12.1	0.950(0.841-1.073)	1.005(0.879-1.149)	0.997(0.871-1.141)
	quartile 3	12.1<PDW≤13.4	1.059(0.942-1.190)	1.160(1.020-1.318) *	1.148(1.009-1.307) *
	quartile 4	PDW>13.4	1.007(0.894-1.134)	1.022(0.897-1.165)	1.020(0.894-1.164)

Abbreviations: isolated systolic hypertension (ISH), isolated diastolic hypertension (IDH), systolic diastolic hypertension (SDH), platelet distribution width (PDW). Analyzed by binary logistic regression.

* *P*<0.05,

** *P*<0.01. No covariates were included in model 1. Age, body mass index (BMI) and waist circumference were included in model 2 as covariates, and age, BMI, waist circumference, creatinine (Cr), uric acid (UA), total cholesterol (TC), triglycerides (TGs), high-density lipoprotein (HDL), low-density lipoprotein (LDL), glucose (GLU) were included in model 3 as covariates.

In addition, MPV is an important factor for hypertension. We performed statistics on the correlation between MPV and PDW in males, females and general participants respectively. MPV and PDW were positively related (Supplementary Table S1).

## Discussion

Previous studies noted that hypertension would lead to an increase in platelet indices, which represented the enhancement of platelet vitality [14,15,23,24]. PDW was considered markers of platelet activation [12,13,19,25,26]. High PDW values may indicate higher production of larger reticulated platelets. Large platelets are more active, release more thromboxane A2 and express more glycoprotein IIb-IIIa receptor, which plays a critical role in coagulation [27]. Thus, changes in platelets reflected by PDW may reveal hypertension status and predict thrombotic risk.

Sex and age differences exist in different hypertension subtypes. Data from a study on hypertension subtypes and stroke risk in rural Chinese adults [28] concluded that IDH was more common in men and that ISH and SDH were more common in women. The ISH group was the oldest on average, followed by the SDH group, and the IDH group had the youngest average age. Li et al. [8] also obtained similar conclusions in a Mongolian cohort from inner Mongolia. Li et al. [29] after investigating rural Mongolian and Han populations, summarized that the prevalence of ISH and SDH increased with advancing age and that the prevalence of IDH decreased beginning at 45 years of age in Han people and 35 years of age in Mongolians. The study also noted that age and female sex were independent risk factors for ISH and SDH in Han people; however, sex was not an independent risk factor for ISH and SDH in Mongolians. Another survey concerning hypertension and metabolic syndrome [30] suggested that whether or not metabolic syndrome was present, the percentage of participants with IDH and SDH gradually decreased and the percentage of participants with ISH gradually increased with age; the change in the prevalence of hypertension subtypes was in line with the findings of the PREVENCION study in Peruvian Andean Hispanics [31]. A high proportion of ISH in the hypertension population led to ISH become the most common hypertensive subtype in persons with metabolic syndrome. Kim et al. [6] noted that three hypertension subtypes appeared more often in men than in women, especially ISH after age adjustment. More interestingly, the general prevalence of the regular patterns of ISH and IDH would be interchanged between men and women beyond 70 years of age when age was taken into account. Compared with IDH, ISH was more associated with cardiovascular events such as coronary artery disease and stroke. However, Ahmed et al. [5] inferred that the prevalence of all hypertension subtypes was higher in women. Being old and being female were important factors in the development of ISH. The likelihood of having ISH and SDH increased with age but decreased for IDH before the adjustment of relevant covariates. The study of Julio et al. [32] focusing on BMI and hypertension hemodynamic subtypes concluded that ISH was present in a small number of hypertension cases among obese men but remained the most common subtype in obese women. A study from Fujimoto et al. [33] mentioned that ISH was more frequently observed in older women. Adeoye et al. [34] concluded that SDH and IDH were more prevalent among women and that the prevalence of ISH was higher among men after investigating hypertension subtypes in Ibadan hypertensive people. Moreover, the middle-aged group (49-59 years) represented a high-incidence age group for the three hypertension subtypes. The data from the Framingham Heart Study [35] supported that more than half of individuals with ISH and IDH were women, and the IDH group was the only group whose mean age was below 50 years (45.7±6.5 years), the youngest of all hypertension subgroups. Being a woman and older were protective factors for new-onset IDH and detrimental factors for new-onset ISH. Another analysis based on the National Health and Nutrition Examination Survey (NHANES) III [36], a survey of middle-aged and elderly US hypertensive individuals, mentioned in both the untreated and inadequately treated hypertensive groups, ISH was the predominant hypertension subtype in individuals over 50 years.

PDW was a risk factor for ISH, and the incidence of ISH increased with PDW quartiles in women. In men, we found no significant relationship between PDW and ISH; however, high PDW might be a risk factor for IDH and SDH. Studies investigating the relationship between PDW and hypertension subtypes are quite rare. A study [18] among different ethnic groups showed that the difference in PDW was insignificant among all subtypes of hypertension in all ethnic groups, and the present study also demonstrated that ISH was associated with abnormal PDW values. However, another study [19] based on the quadratic inference function method showed that PDW was negatively associated with SBP and that there were no associations between PDW and DBP. In addition to different study methods, the lack of a classification of hypertension subtypes and the longitudinal study design were significant differences compared to our cross-sectional study. In addition to the above studies, we may be able to learn from other analogous studies, such as studies concerning pulmonary arterial hypertension and platelet indices, which might help us to comprehend the relationship between elevated blood pressure and PDW. A survey [24] from Chinese Academy of Medical Sciences and Peking Union Medical College noted that patients with idiopathic pulmonary hypertension had higher PDW than healthy controls, and PDW was significantly correlated with pulmonary arterial pressure. Similarly, another study [37] conducted with 37 participants with congenital heart disease with pulmonary arterial hypertension (APAH-CHD) and 43 healthy controls showed that PDW was higher in APAH-CHD participants than in controls and that the PDW of individuals who died was higher than that of surviving individuals during follow-up. PDW was positively related to mean pulmonary artery pressure. Some possible mechanisms were explained. Some hypertension-related inflammation and immune processes cause platelet activation. Elevated pulmonary arterial blood pressure leads to vascular endothelial dysfunction, which might cause platelet activation and local thrombosis. Activated platelets are more likely to adhere to local injured pulmonary artery endothelium and promote the formation of thrombi in hypertension patients. Moreover, growth factors and cytokines released by activated platelets might play critical roles in the remodeling of pulmonary vessels, which may be linked to the progression of elevated blood pressure in the pulmonary artery. Active platelets contain greater PDW values, and the coagulation of active platelets or local thrombosis would further increase the abnormality of PDW [13,27]. Moreover, Routledge et al. [38] supposed that women with untreated stage 1 hypertension had greater endothelial dysfunction than their male counterparts, but the phenomenon in women did not change with the presence or absence of sex hormone protection. However, estrogen is a protective factor against hypertension [39]. The prevalence of hypertension was lower in premenopausal women than in men of the same age [40]. After menopause, the prevalence of hypertension in women would increases [41]. Although the relationships of different hypertension subtypes with age and sex are controversial, a considerable number of studies [5,33,35,29] agree with the view that being female and being old play pivotal roles in the development of ISH. Another study [42] from Sheikh et al. showed that platelet activation decreased after hormone replacement therapy among postmenopausal women. Our data showed that the prevalence of ISH exceeded half of the population of individuals over 60 years for the first time in females, and the mean age of women with ISH was 63.15 years, which was older than the usual menopause age. Thus, the lack of estrogen may lead to a different prevalence of hypertension subtypes between men and women. Estrogen deficiency seems to play a crucial role in the inconsistent results between different sexes.

## Limitations

Our data comes from Tianjin Medical University General Hospital Health Management Center. Participants are usually well-educated and attached importance to self-health management, which may cause selection bias. Blood samples were obtained only once without repeated validations and heart and vascular system related and hormone-related parameters were not acquired, which will be collected in our future studies. This is a cross-sectional study, and a long-term follow-up record should be established to trace the relationship between PDW and hypertension.

## Supplementary Material

Supplementary Table S1

## Data Availability

The original data used to support the findings of this study are available from the corresponding author upon request.

## Abbreviations

BMI: body mass index
Cr: creatinine
DBP: diastolic blood pressure
GLU: glucose
HDL: high-density lipoprotein
IDH: isolated diastolic hypertension
ISH: isolated systolic hypertension
LDL: low-density lipoprotein
MPV: mean platelet volume
PDW: platelet distribution width
SBP: systolic blood pressure
SDH: systolic diastolic hypertension
TC: total cholesterol
TGs: triglycerides
UA: uric acid
