# Supplementary material for: The different associations between platelet distribution width and hypertension subtypes in males and females
Source: Biosci Rep. 2020 Nov 19;40(11):BSR20201747. doi: 10.1042/BSR20201747 (PMC7677823; doi:10.1042/BSR20201747)
Supplement: Supplementary Table S1 [file BSR-2020-1747_supp.pdf]

Table S1. Pearson correlation of MPV and PDW

	Male	Female	General
Correlation	0.676^{**}	0.704^{**}	0.687^{**}

Abbreviations: mean platelet volume (MPV), platelet distribution width (PDW). $^{**}p<0.01$.
